# Can a posterior approach effectively heal thoracic and lumbar tuberculosis? Microbiology outcomes of the operative area

**DOI:** 10.1186/s13018-019-1063-7

**Published:** 2019-01-22

**Authors:** Chen Zhao, Xiaobing Pu, Qiang Zhou, Xingzhou Huang, Chengmin Zhang, Lei Luo, Zehua Zhang, Tianyong Hou, Fei Luo, Fei Dai, Jianzhong Xu

**Affiliations:** 1 Department of Orthopedics, Southwest Hospital, The Army (Third Military) Medical University, GaoTanYan 29, Chongqing, 400038 China; 2 Department of Orthopedic Surgery, No.4 West China Teaching Hospital, Sichuan University, Chengdu, 610000 Sichuan China

**Keywords:** Spinal tuberculosis, Anterior approach, Posterior approach, Microbiology outcomes, Debridement

## Abstract

**Background:**

There was a controversy about surgery approach of thoracic and lumbar tuberculosis (TB) treatment. The aim of this study was to compare the microbiology outcomes of the drainage liquid and the clinical outcomes of a posterior and anterior approach in the treatment of thoracic and lumbar TB.

**Materials and methods:**

A total of 105 patients were enrolled in this prospective study from February 2011 to September 2015. Patients were divided into two groups: group A (51 patients, posterior approach surgery) and group B (54 patients, anterior approach surgery). Intraoperative TB samples were sent for Mycobacterium tuberculosis culture (MTBC). Drainage fluid was postoperatively collected for polymerase chain reaction (PCR), acid-fast strains (AFS), MTBC, and DNA molecular detection (DNAMD) analyses. Compare the drainage liquid positive rate of the two groups and estimate relationship between the positive results of drainage fluid and the lesion region. In addition, the clinical outcomes including the bony fusion, relapse rate, complications, and neurological status were collected.

**Results:**

There was no significant difference in the positive rate of AFS, PCR, DNAMD, MTBC, or any positive rate (APR) of drainage liquid between the two groups (*P* > 0.05). In both groups, the MTBC-positive rate of postoperative drainage fluid was significantly lower than that of the intraoperative sample (*P* < 0.01). There was no significant relationship between APR and the lesion region (*P* > 0.05). All the patients had at least 2 years of follow-up, with an average of 34.4 ± 15.8 months. There were four patients in group A and two patients in group B who had recurrent spine TB, and the rest of the patients had fusion in the surgical area. There was no significant difference in the incidence of TB recurrence or other complications between the two groups (*P* > 0.05). All the patients with neurological dysfunction had improved after surgery.

**Conclusion:**

Compared with anterior approach surgery, posterior approach surgery had equal effectiveness of debridement. The two kinds of surgery can effectively clear the lesions surrounding the spine and heal thoracic and lumbar TB.

## Background

Thoracic and lumbar tuberculosis (TB) is the most common type of spinal TB [1]. Surgical procedures are considered by spinal surgeons when patients have neurological deficiency, spinal deformity, or instability. Lesion debridement is the basis of successful surgical treatment of thoracolumbar TB.

Spinal TB undermines the anterior and middle column in most cases, so anterior debridement has been the classic surgical treatment of thoracic and lumbar TB [2]. Anterior debridement and bone grafting combined with anterior or posterior fixation have been widely used and obtained good clinical outcomes [3–7]. However, there are some shortfalls of anterior approach surgery, such as complex anatomy, difficult to deal with multiple segmental lesions, and severe kyphosis cases. Moreover, anterior-combined posterior surgery would increase the surgical trauma, time, and complications [8].

In recent years, posterior approach debridement combined with bone graft and pedicle screw fixation has been used to treat thoracic and lumbar TB. Early posterior surgery was mostly used to treat mild cases [9, 10]. As the technology kept improving, it was gradually applied to serious cases [4, 5, 11, 12]. The main advantage of the single posterior approach was that the same incision was used to complete lesion debridement, orthopedic bone grafting, and internal fixation.

It has been reported in clinical studies that the same effect can be obtained in posterior surgery compared with traditional anterior surgery or anterior combined with posterior approach surgery [4, 5, 8]. However, some surgeons still doubted the efficiency of debridement, because the posterior approach sometimes could not be operated under direct vision.

The reason for the study design was that TB microbiology testing of the operative area could indirectly response residual TB in the operative area. Combined with the clinical results, such testing can helpfully evaluate the effectiveness of debriding the lesion.

## Materials and methods

### Inclusion criteria

Inclusion criteria include (1) patients with active thoracic and lumbar TB; (2) both anterior and posterior surgery can achieve lesion debridement, bone graft fusion, and stable spine. Surgical indications include neurological impairment, spinal deformity or instability, and failed conservative treatment; and (3) aged 18–70 years old.

### Exclusion criteria

Exclusion criteria include (1) recurrent thoracolumbar TB; (2) severe osteoporosis; and (3) combination with spinal tumors, lumbar disc herniation, spondylolisthesis, or other conditions, which affect the clinical efficacy.

### General clinical data

This study is a prospective clinical study and has been approved by the Southwest Hospital Ethics Committee. From February 2011 to September 2015, 113 patients were enrolled in the study. All patients had signed the consent form. The patients were divided into group A (single posterior approach surgery group) and group B (single anterior approach surgery group). At follow-up time, eight patients were lost. A total of 105 patients were included in the statistical analysis. Clinical data are shown in Table 1. The lesion regions of the two groups are listed in Table 2. There was no difference between the two groups before surgery (*P* > 0.05).

**Table 1:** General data

	Group A	Group B	*P*
Patients (no.)	51	54	
Gender (male/female)	27/24	19/35	> 0.05
Age (Y/O)	36.5 ± 13.9	37.6 ± 13.2	> 0.05
Neurological deficits (no.)	19	29	> 0.05
Average lesion segments	1.3 ± 0.7	1.2 ± 0.6	> 0.05
Lesion region (no.)			
Thoracic	16	18	> 0.05
Thoracolumbar	18	14	> 0.05
Lumbosacral	17	22	> 0.05

There was no statistical difference in the general data between the two groups

**Table 2:** Lesions region

	Group A	Group B	*P*
Anterior vertebral	32	39	> 0.05
In the spinal canal	25	36	> 0.05
Bilateral paravertebral	25	31	> 0.05
Beyond the lesion vertebral	18	25	> 0.05

There was no statistical difference in the lesion region between the two groups

### Preoperative management

After admission, all patients completed blood tests: routine blood, liver and kidney function, erythrocyte sedimentation rate (ESR), and C-reactive protein (CPR) as well as spinal X-ray, three-dimensional (3D) computed tomography (CT) (Somatom Definition, SIEMENS Corp., Germany) reconstruction, and magnetic resonance imaging (MRI) (Magnetom Avanto 1.5 T, SIEMENS Corp., Germany) examinations for bone destruction and cold abscess. A clinically standard chemotherapy regimen was administered for at least 2 weeks before the operation, which consisted of isoniazid 300 mg per os (PO) every day (QD), rifampin 450 mg PO QD, pyrazinamide 500 mg PO three times daily (TID), and ethambutol 750 mg PO QD. Patients also received levofloxacin 200 mg intravenously (IV) twice daily (BID) during hospitalization. The preoperative treatment also included nutritional support and correction of anemia and hypoproteinemia.

### Surgical technique

#### Posterior approach surgery

**Fig. 1:**
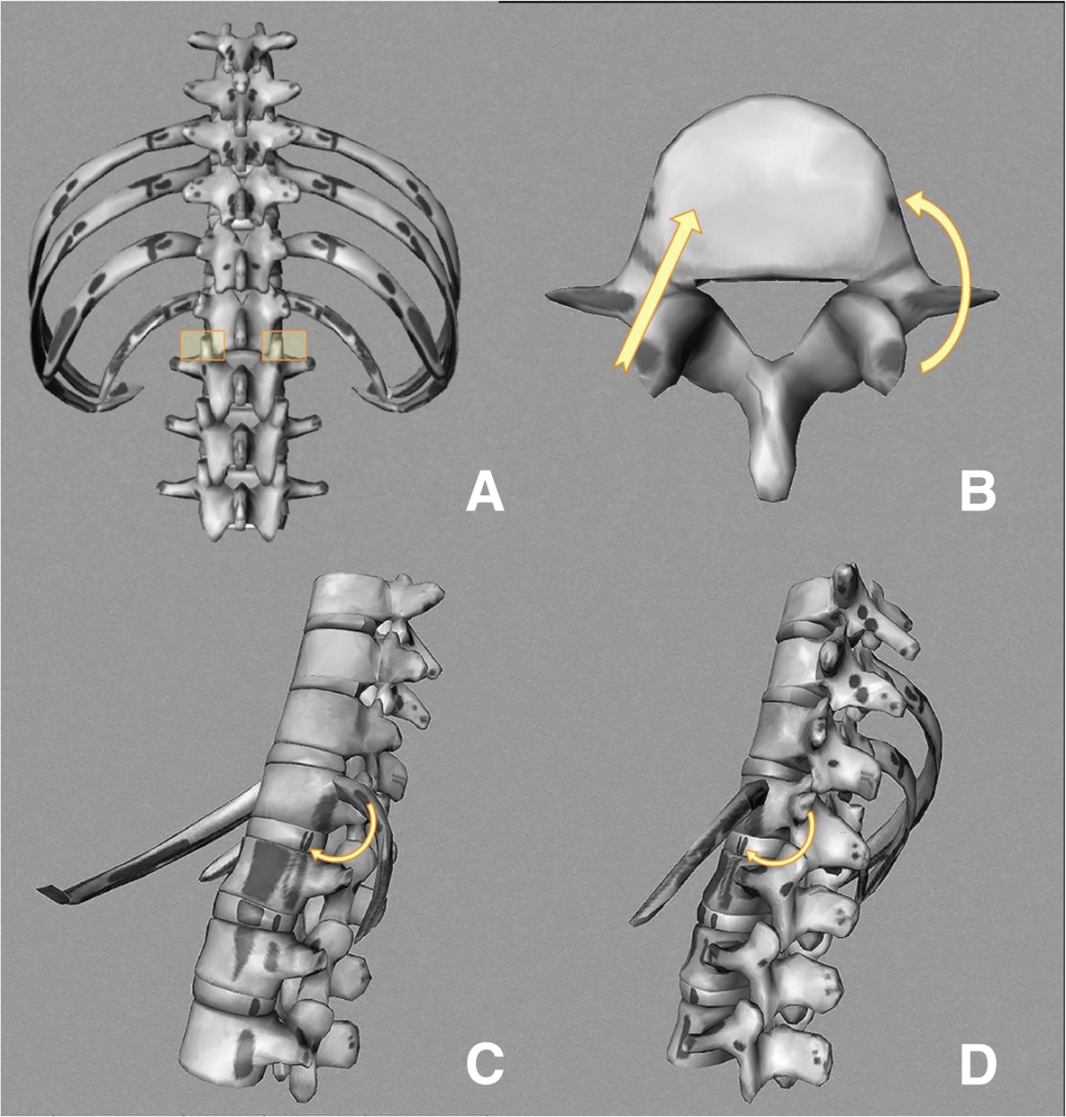
**a**, **b** The middle line posterior approach was used to expose the bilateral lamina, and the unilateral or bilateral facet joint and transverse processes were removed. By transforaminal and paravertebral approach, the lesion around the vertebrae was completely debrided. **c**, **d** The transversectomy process approach was performed in the thoracic area, and the extraperitoneal approach was performed in the lumbar area. Remove the same side transverse process and rib. The lesion was completely debrided by lateral of vertebral body

After standard general anesthesia and sterilization, the patient was placed in the prone position, and the middle line posterior approach was used to expose the bilateral lamina. According to the lesion range, the unilateral or bilateral facet joint and transverse processes were dissected. By transforaminal and paravertebral approach, the lesion around the vertebrae was completely removed (Fig. 1). The surgical area was irrigated with large quantities of hydrogen peroxide and normal saline (> 3000 ml). According to the size of the bone defects, pedicle screws were implanted and a cage or titanium mesh full of autogenous bone was chosen to perform interbody support and fusion. Allogeneic bone was used to reconstruct lamina, then posterolateral bone graft and fusion was performed. The drain was placed in the surgical area and the incision sutured.

#### Anterior approach surgery

**Fig. 2:**
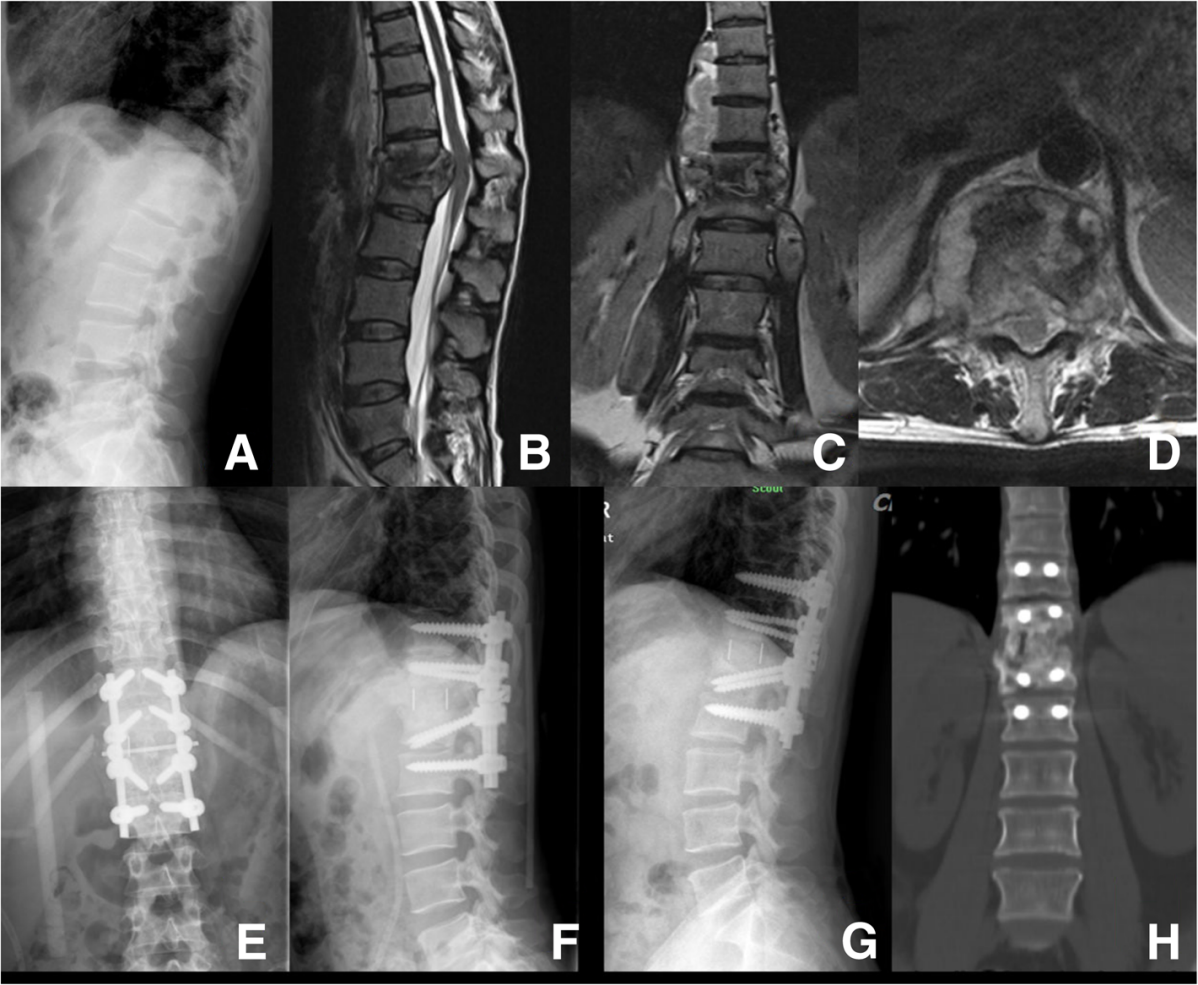
A 38-year-old female patient in group A, and the diagnosis was T12-L1 TB. **a**–**d** Preoperative X-ray and MRI. The MTB lesions at anterior vertebral, bilateral paravertebral, spinal canal, and beyond the vertebral lesion. **e**, **f** 1 week postoperative X-ray. **g**, **h** 1 year postoperative X-ray and CT scan

**Fig. 3:**
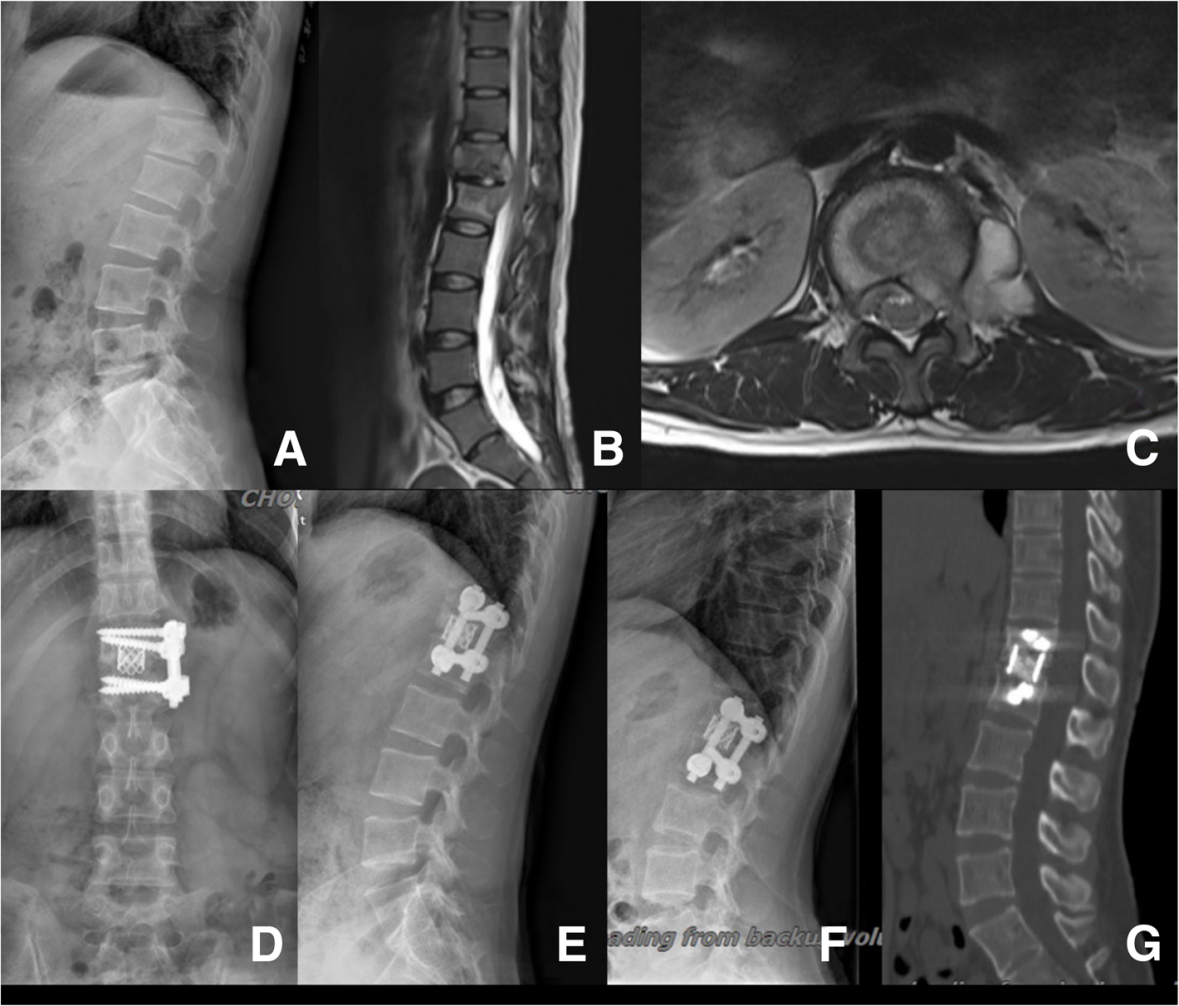
A 30-year-old female patient in group B, and the diagnosis was T12-L1 TB. **a**–**c** Preoperative X-ray and MRI. The MTB lesions at unilateral paravertebral, spinal canal, and not beyond the vertebral lesion. **d**, **e** 1 week postoperative X-ray. **f**, **g** 1 year postoperative X-ray and CT scan

After standard general anesthesia and sterilization, the patient was placed in the lateral position. The worse lesion side had been positioned to the upper side. The transversectomy process approach was performed in the thoracic area, and the extraperitoneal approach was performed in the lumbar area, in order to access the lesion (Fig. 2). After complete debridement of TB tissue, the surgical area was copious irrigated. A cage or titanium mesh was used to support interbody, then internal fixation and bone graft was performed. The drain was placed in the surgical area like posterior approach surgery (Fig. 3).

All surgeries were performed by the same team of surgeons. Isoniazid 300 mg and rifamycin 500 mg were mixed with bone graft in both groups.

### Drainage management

A drainage catheter was placed in the surgical area, specifically on the surface of bilateral lamina in patients of group A and in front of or lateral to the vertebral body in group B. At 48 h and 96 h after the surgery, all drainage fluid was collected, centrifuged, and sent for testing. Samples were kept away from light during transportation. Acid-fast staining (AFS) (Staining fluid ST1705, Pang Tong Medical Devices Company, Chongqing, China) and polymerase chain reaction (PCR) (MTB-DNA diagnostic kit, QIAGEN Corp., Germany) analyses were performed at 48 h postoperatively, and DNA molecular detection (DNAMD) (DNA chip with CapitalBio TM microarray, CapitalBio Corp., Beijing, China) and Mycobacterium tuberculosis culture (MTBC) were performed at 96 h postoperatively. MTBC included rapid culture with a BACT/MGIT 960 system (Becton, Dickinson and Company, New Jersey, USA) and Lowenstein-Jensen culture (Mycobacteria L-J Culture Medium, Encode Medical Engineering Company, Zhuhai, China).

### Postoperative management

Tissue specimens taken from the TB lesion were cultured, and the method was the same as the drainage. For all patients, the vital signs and the sensation and motion of both lower extremities were routinely tested, and the treatments for infection prevention, anti-TB drugs, nutritional support, and correction of anemia, and hypoproteinemia were administered. The patients were required to wear the brace for 3–6 months when doing out-of-bed activities and to take anti-TB drugs for 12–18 months. All patients returned to the hospital for X-ray, clinically examination, and 3D-CT reconstruction or MRI examination as necessary at 3, 6, 12, 18, and 24 months after surgery, then once a year thereafter. When the patient showed bone graft fusion in the CT and non-TB lesion in the MRI and ESR, CRP was considered normal, as it showed the healing of TB, and the patient could stop using anti-TB drugs.

### Evaluation of microbiology and clinical outcomes

All the microbiology outcomes were recorded and statistically analyzed. In one case, rapid culture or Lowenstein-Jensen was positive, and we recorded this case as MTBC positive. If any tests of AFS, PCR, DNAMD, or MTBC of drainage were positive, we recorded them as any positive rate (APR). Bony fusion, complications in the perioperative and follow-up periods, and neurological status were observed. Neurological status was evaluated using the American Spinal Injury Association (ASIA) impairment scale, and bony fusion was evaluated by CT-3D reconstruction.

### Statistical analysis

SPSS 19 was used for data analysis. The average age and lesion segments were compared by Student’s *t* test. The other general data, lesion region, proportion of positive cases, incidence complications, neurological function, and recurrence rate were compared by chi-square test. The logistic regression was used to estimate relationship between the APR and the lesion region.

An alpha value of 0.05 was considered significant.

## Results

### Microbiology outcomes

The positive rates of intraoperative lesion cultures in groups A and B were 66.7% and 63.6%, respectively. Microbiology outcomes of drainage fluid are shown in Table 3. Overall, more than 25% of the subjects had at least one positive reading. There were no significant differences between the two groups in the positive rate of drainage test, APR, and intraoperative TB tissue MTBC (*P* > 0.05). In both groups, the MTBC-positive rate of postoperative drainage fluid was significantly lower than that of the intraoperative sample (*P* < 0.01).

**Table 3:** Drainage fluid microbiology results

	Group A (%)	Group B (%)	*P*
Any positive rate	33.3	27.8	> 0.05
Acid-fast staining	0	0	
PCR	27.5	17.7	> 0.05
Gene chip	7.8	7.4	> 0.05
Culture	7.8	9.3	> 0.05

There were no significant differences in the APR (any positive rate) and each test between the two groups

The logistic regression results were showed in Table 4. The logistic regression showed that there was no significant relationship between the APR of drainage fluid and the lesion region in both groups (anterior vertebral, bilateral paravertebral, spinal canal, and beyond the vertebral lesion).

**Table 4:** The relationship between the any positive rate of drainage fluid and the lesion region

	*P* (group A)	*P* (group B)
Anterior vertebral	> 0.05	> 0.05
In the spinal canal	> 0.05	> 0.05
Bilateral paravertebral	> 0.05	> 0.05
Beyond the lesion vertebral	> 0.05	> 0.05

There was no significant relationship between the APR (any positive rate) of drainage fluid and the lesions region in both groups

### Clinical outcomes

The average follow-up duration was 34.4 ± 15.8 months (range 24 to 79 months). Ninety-nine (95.2%) patients were healed by the first surgery and showed bone fusion in CT scan. There were no internal fixation-related complications. Four patients from group A had recurrence of spinal TB. Their clinical symptom was back pain, and MRI showed there is TB lesion in the surgical area at 3, 6, 18, and 36 months postoperatively. Two of the patients were successfully treated with a posterior approach re-operation, and another two were successfully treated with conservative management. Two patients in group B had recurrence of spinal TB at 1 and 48 months postoperatively. They were treated by conservative treatment and anterior combined with posterior surgery, respectively. There was no significant difference in the rate of recurrence between the two groups (*P* > 0.05).

In group A, there were three cases of cerebrospinal fluid leakage: one case with pulmonary infection, one case with respiratory failure, and one case with acute heart failure. In group B, there were three patients with pain in the corresponding innervation area and muscle strength decrease, six cases of pleural effusion, and one case of pulmonary infection. All of them were cured after conservative treatment. There was no significant difference in the incidence of complications between the two groups (*P* > 0.05). There was no case of tuberculous meningitis in either group.

In group A, there were 19 (two cases with B grade, 17 with D grade) cases of concomitant nerve injury compared with 29 (one case with B grade, one with C grade, 27 with D grade) cases in group B. There was no statistical difference in preoperative nerve function between group A and group B (*P* > 0.05). At the final follow-up, all patients’ neurological function had improved. One case with grade B in group A and group B returned to grade D respectively, and the other patients recovered to grade E. There were statistically significant differences between the preoperative and final follow-up neurological function in each group (*P* < 0.01). And there was no statistical difference in the final follow-up nerve function between the two groups (*P* > 0.05).

## Discussion

There was a controversy about the posterior surgery because its partial operation is not under direct vision, which could affect the lesion removal effectiveness. The debridement of TB lesions plays an important role in surgery and affects clinical outcomes. Most surgeons believe the lesion should be removed cleanly. However, there is no study about microbiology outcomes of posterior approach lesion removal effectiveness.

Presently, there are various methods for MTB testing. The AFS smear test is a classic testing method for MTB, which sensitivity was 25–75% [13]. PCR is a DNA amplification-based biological diagnosis technique and highly applicable in the rapid diagnosis of MTB [14, 15]. CapitalBio (TM) microarray is a kind of MTB-DNAMD, which can simultaneously detect 17 kinds of mycobacteria by detecting specific sequences in 16S rRNA and identifying whether these mycobacteria are rifampicin and isoniazid resistant. MTB-DNAMD has 93.55% sensitivity [16] and 100% specificity for the species identification of MTB [17, 18]. MTBC includes many methods, such as Lowenstein-Jensen culture and rapid culture and is the gold standard for MTB testing, which has a positive rate of 48.6–80% [13]. Considering MTB concentration in the operative area was decreased after the operation, we used multilevel microbiology tests mentioned and centrifuged the samples before testing to improve the results accuracy.

MTB microbiology outcomes of the operative area could indirectly response residual TB in the operative area. In our study, the positive rates of the drainage MTBC were significantly lower than those of the intraoperative lesion in both groups. The positive rates of AFS, PCR, and DNAMD microarray of drainage fluid were all markedly lower than those of TB focus, with decreases of 32%, 98%, and 74.5%, respectively [13, 16, 19]. The results showed that the surgical area TB concentration had dropped significantly. Research has shown that MTB AFS usually requires approximately 10^4^ bacilli/ml to produce positive results [20, 21]. Most specimens that were AFS negative were also negative in Lowenstein-Jensen culture, and even if they were positive, the MTB < 100 colonies [22, 23]. PCR is positive in specimens of at least 10–50 tubercle bacilli [24]. This study showed that there was negative AFS smearing under the microscope and positive rates of PCR < 30% in both groups. Therefore, we believe that postoperative TB concentration of the surgical area < 10^4^/ml, with most even less than 10–50 tubercle bacilli, which could reduce TB reproduction and provide a local environment for the cure of TB.

Surgical treatment of spinal TB should be individualized because the anterior and posterior approaches have their own characteristics [25]. The anterior approach is easy to address the lesion and deal with the same side psoas or iliaca fossa abscess. The posterior approach anatomy is simple and provides convenient to debride the lesion which extended in the spinal canal. There was potential risk that debridement may not complete in both of approaches, because operation has to be undertaken in non-sight view when the lesions were in contralateral vertebral for anterior approach or in front of vertebral for posterior approach. This study found that there was no significant relation to APR of drainage with lesion region and 95.2% patients were healed by the first surgery. Therefore, we believe that both approaches could clear the lesions surrounding the thoracic and lumbar vertebrae to promote TB healing. In addition, there was no significant difference in APR, the positive rate of each test, and the TB recurrence rate between the two groups. All the patients with neurological dysfunction had improved after the surgery. The results showed that the posterior and anterior approaches have the same lesion removal efficiency.

Spinal TB treatment was comprehensive. There were various factors related to clinical outcomes, such as the effectiveness of lesion clearance, the stability of spine, the sensitivity of anti-TB drugs, and the patient’s own immunity. Our results showed that most of the drainage test positive or MTBC-positive cases were cured. Then, we believe that debridement can clear most of the MTB and improve circulation of the tissue to promote healing, but not the clearance of all MTB. The rest of the MTB can be controlled by anti-TB drugs and comprehensive treatment after surgeries.

## Conclusion

Posterior and anterior surgeries had the same lesion removal efficiency. The two kinds of surgery can effectively clear the lesions surrounding the spine and heal thoracic and lumbar TB.

## Abbreviations

3D: Three-dimensional
AFS: Acid-fast strains
APR: Any positive rate
ASIA: American Spinal Injury Association
BID: Twice daily
CPR: C-reactive protein
CT: Computed tomography
DNAMD: DNA molecular detection
ESR: Erythrocyte sedimentation rate
IV: Intravenously
MRI: Magnetic resonance imaging
MTBC: Mycobacterium tuberculosis culture
PCR: Polymerase chain reaction
PO: Per os
QD: Every day
TB: Tuberculosis
TID: Three times daily
